# Heerfordt Syndrome Complicated by Bilateral Simultaneous Facial Palsy, PTH-Independent Hypercalcemia, and Bilateral Obstructive Acute Kidney Injury in the Absence of Thoracic Disease: A Case Report and Narrative Review

**DOI:** 10.3390/reports9030225

**Published:** 2026-07-15

**Authors:** Khaled Abdulwahab Amer, Nawaf Ibrahim Al Shuqayfah, Mohammad Abdallah Alhakamy, Abdullah Jaber Alasiri

**Affiliations:** 1 Department of Internal Medicine, Aseer Central Hospital, Aseer Health Cluster, Abha 62523, Saudi Arabia; nawaf-2468-24@hotmail.com; 2 Division of Nephrology, Department of Internal Medicine, Aseer Central Hospital, Aseer Health Cluster, Abha 62523, Saudi Arabia; dr.alfie2014@gmail.com (M.A.A.); aj.asiri@yahoo.com (A.J.A.)

**Keywords:** sarcoidosis, Heerfordt syndrome, uveoparotid fever, hypercalcemia, obstructive uropathy, acute kidney injury, bilateral facial palsy, calcitriol, case report

## Abstract

**Background and Clinical Significance**: Heerfordt syndrome (uveoparotid fever) is an uncommon extrapulmonary expression of sarcoidosis defined by parotid enlargement, uveitis, low-grade fever, and facial nerve palsy. It is identified in fewer than 1% of patients with biopsy-confirmed sarcoidosis, and the co-occurrence of bilateral seventh-nerve palsy with calcitriol-driven obstructive kidney injury has been reported only sporadically. Recognizing this metabolic-renal phenotype is clinically important because it is readily reversible yet easily missed when the chest radiograph is normal. **Case Presentation**: A 30-year-old man presented with four months of progressive bilateral parotid swelling, sicca symptoms, and intermittent blurred vision, preceded by a self-limited episode of bilateral simultaneous peripheral facial weakness; renal colic developed one month before admission. Evaluation showed parathyroid-hormone-independent hypercalcemia (peak corrected calcium 13.1 mg/dL), a serum angiotensin-converting enzyme level above 100 U/L, and acute kidney injury (creatinine 2.1 mg/dL) caused by bilateral upper-ureteric calculi with hydronephrosis. Posterior uveitis was confirmed ophthalmologically. Chest radiography and high-resolution thoracic computed tomography were unremarkable, with no hilar lymphadenopathy. Parotid gland biopsy demonstrated non-caseating epithelioid granulomas. Bilateral ureteric stenting and oral prednisolone 40 mg daily produced rapid normalization of serum calcium, recovery of renal function, and regression of parotid enlargement. **Conclusions**: This report characterizes a phenotype that combines bilateral simultaneous facial-nerve involvement, calcitriol-mediated hypercalcemia, and obstructive nephrolithiasis without coexistent pulmonary disease. It argues for early consideration of sarcoidosis whenever hypercalcemia, parotid enlargement, and cranial neuropathy occur together, and reinforces the reversibility of sarcoidosis-related renal injury when corticosteroids are introduced promptly.

## 1. Introduction and Clinical Significance

Sarcoidosis is a granulomatous disorder of unknown etiology in which dysregulated cellular immunity drives the formation of compact, non-caseating epithelioid granulomas across multiple organs. The lungs and intrathoracic lymph nodes are involved in roughly 90% of patients, but virtually any tissue can be affected, and the clinical phenotype varies widely between geographic regions and ethnic groups. Reported incidence estimates range from 3 to 50 per 100,000 person-years, with the highest figures coming from northern European and African American populations [1,2].

Christian Frederik Heerfordt described the constellation of febrile parotid enlargement, uveitis, and facial nerve palsy in 1909, more than a decade before sarcoidosis was conceptualized as a unifying disease entity. Waldenström subsequently linked the syndrome to the wider spectrum of sarcoidosis, and the eponym “Heerfordt–Waldenström syndrome” remains in use [3,4]. In contemporary case series the complete tetrad—fever, parotid enlargement, uveitis, and facial-nerve palsy—is encountered in only about 10–15% of patients presenting with Heerfordt syndrome, corresponding to well under 1% of all sarcoidosis cohorts; the true frequency is almost certainly higher because incomplete or sequential presentations are easily overlooked [4]. Facial nerve palsy occurs in about 5% of sarcoidosis cases overall and is the most common cranial neuropathy in neurosarcoidosis [5].

Renal manifestations of sarcoidosis cluster into three broad categories: disorders of calcium homeostasis (hypercalcemia, hypercalciuria, nephrolithiasis, and nephrocalcinosis), granulomatous interstitial nephritis, and, less commonly, glomerular or vascular disease. Subclinical kidney involvement may be present in up to half of patients with chronic disease, although overt renal failure is far less common [6,7]. Hypercalcemia in sarcoidosis is driven by extrarenal conversion of 25-hydroxyvitamin D to 1,25-dihydroxyvitamin D by 1α-hydroxylase (CYP27B1) expressed in granuloma-resident macrophages. Unlike the renal isoform, this enzyme escapes the normal negative feedback exerted by parathyroid hormone, calcium, and fibroblast growth factor-23, and the resulting calcitriol surge enhances intestinal calcium absorption and bone resorption [8,9].

We describe a young man whose presentation of Heerfordt syndrome was dominated by metabolic and renal complications rather than pulmonary disease, including bilateral simultaneous facial palsy and acute kidney injury from bilateral stone-related ureteric obstruction. The case is accompanied by a focused narrative review of the clinical phenotypes, differential diagnosis, and therapeutic considerations relevant to this rare overlap.

## 2. Case Presentation

### 2.1. History

A 30-year-old man with a childhood diagnosis of eczema and no history of tobacco use, occupational dust exposure, recent travel, or known autoimmune illness was referred to our internal medicine service. He had experienced painful bilateral parotid swelling for four months, with no improvement after two short courses of empirical antibiotics, accompanied by xerostomia, ocular dryness, and intermittent blurred vision. Two months before admission he had a self-limited, roughly two-week episode of bilateral simultaneous peripheral facial weakness with incomplete eye closure, drooling, and loss of forehead furrowing on both sides; the weakness resolved spontaneously before referral. One month before admission he developed bilateral flank pain, intermittent colicky discomfort, and dysuria without gross hematuria. There was no fever, weight loss, night sweats, arthralgia, skin lesions, productive cough, exertional dyspnea, or gastrointestinal symptom. His father had advanced diabetic–hypertensive kidney disease requiring hemodialysis; there was no family history of sarcoidosis, autoimmunity, or nephrolithiasis.

### 2.2. Physical Examination

On admission the patient was alert, oriented, and hemodynamically stable. Both parotid glands were diffusely enlarged, firm, and tender, with mild overlying erythema; there was no fluctuation, and no discharge could be expressed from the Stensen ducts. There was no peripheral lymphadenopathy, hepatosplenomegaly, peripheral edema, or cutaneous involvement (no erythema nodosum, lupus pernio, or plaque lesions). Cranial-nerve examination was normal, the antecedent facial weakness having resolved, and the remainder of the neurological examination, including reflexes and sensation, was unremarkable. Cardiac, respiratory, and abdominal examinations were normal.

### 2.3. Laboratory Findings

Initial biochemistry revealed acute kidney injury (serum creatinine 2.1 mg/dL; urea 64 mg/dL) and severe hypercalcemia, peaking at 13.1 mg/dL with normal serum albumin. Intact parathyroid hormone was suppressed at 3.4 pg/mL. Serum angiotensin-converting enzyme exceeded the upper detection limit of the assay (>100 U/L; reference 8–52 U/L). 25-hydroxyvitamin D was insufficient at 22 ng/mL; the 1,25-dihydroxyvitamin D assay was unavailable at our institution during the admission, which we acknowledge as a limitation. Inflammatory markers were mildly elevated (ESR 19 mm/h, CRP 10 mg/L). A full autoimmune panel (antinuclear antibodies, anti-Ro/SSA, anti-La/SSB, c-ANCA, and p-ANCA) was uniformly negative. Complement (C3, C4) and immunoglobulin G were within reference limits. Screening for human immunodeficiency virus, hepatitis B surface antigen, and hepatitis C antibody was non-reactive, as was an interferon-gamma release assay for Mycobacterium tuberculosis. Urinalysis showed sterile pyuria (>100 leukocytes per high-power field, with negative cultures on four separate occasions), microscopic hematuria, dipstick proteinuria, and hyaline casts; quantified 24 h urinary protein excretion was 595.9 mg. Complete laboratory results are summarized in Table 1 and Table 2.

### 2.4. Imaging Studies

Posteroanterior chest radiography demonstrated clear lung fields, a normal cardiomediastinal silhouette, and no hilar prominence or parenchymal infiltrate (Figure 1). High-resolution thoracic computed tomography was likewise negative for parenchymal disease, mediastinal adenopathy, or bilateral hilar lymphadenopathy. This was a notable finding, given that more than 90% of sarcoidosis cases harbor some thoracic radiographic abnormality at diagnosis.

Non-contrast computed tomography of the abdomen and pelvis revealed obstructive calculi within the proximal third of both ureters, measuring 8 mm on the right and 6 mm on the left, with mild upstream pelvicalyceal dilatation. Multiple small non-obstructing parenchymal stones (2–4 mm) were present bilaterally (Figure 2). Renal ultrasound corroborated grade II–III hydronephrosis with diffuse parenchymal hyperechogenicity, fair corticomedullary differentiation, and fullness of both collecting systems; an incidental left adrenal nodule (1.3 cm, 4 Hounsfield units) was consistent with a benign, most likely non-functioning adenoma on the basis of its imaging characteristics alone (size and low attenuation of 4 HU); dedicated biochemical screening for hormonal excess (an overnight 1 mg dexamethasone-suppression test and plasma metanephrines) was not pursued, as there were no clinical or biochemical features to suggest a functioning lesion, so the “non-functioning” designation rests on imaging characteristics alone. Neck ultrasound showed diffuse parotid enlargement with heterogeneous echotexture and intraparenchymal lymph nodes, together with subcentimetric cervical and submandibular nodes with preserved fatty hila. Dual-energy X-ray absorptiometry of the lumbar spine and bilateral hips returned T-scores within the normal range, arguing against chronic skeletal calcium mobilization. Ophthalmological assessment identified bilateral posterior uveitis with mild vitritis but no granulomatous keratic precipitates.

### 2.5. Histopathology

Open biopsy of the right parotid gland was chosen over bronchoscopic or skin biopsy because superficial salivary disease offered an accessible, high-yield target in the absence of radiographic thoracic involvement. Because of the antecedent (albeit resolved) bilateral facial weakness, the specimen was taken from the superficial lobe only through a standard modified Blair approach, with intraoperative identification and preservation of the facial-nerve trunk and its peripheral branches; no new facial weakness followed the procedure. Microscopy demonstrated well-formed, compact, non-caseating epithelioid granulomas with scattered multinucleated giant cells distributed in the interlobular stroma. There was no necrosis, fibrinoid degeneration, or vasculitic change. Special stains for acid-fast bacilli (Ziehl–Neelsen) and fungal elements (Grocott methenamine silver and periodic acid–Schiff) were negative, and tissue culture for mycobacteria and fungi yielded no organisms. In the appropriate clinical setting, and after exclusion of competing granulomatous diseases, this pattern was diagnostic of sarcoid involvement of the parotid gland.

### 2.6. Management and Outcome

Bilateral retrograde double-J ureteric stenting was performed by the urology service for the obstructive component of the renal injury; urine output increased and serum creatinine declined within the first 48 h. Once histological confirmation was obtained, oral prednisolone was started at 40 mg daily (approximately 0.5 mg/kg/day). Serum calcium reached the normal range within seven days of starting corticosteroids, creatinine settled at 1.21 mg/dL by day fourteen, the parotid glands visibly regressed within two weeks, and serum ACE began to trend downward. The patient was discharged on a tapering steroid regimen with planned nephrology and ophthalmology follow-up. At three-month review he remained in clinical and biochemical remission, with a creatinine of 1.05 mg/dL, normal calcium, and resolution of posterior uveitis. The clinical course is summarized in Figure 3.

## 3. Discussion

This patient drew together a striking and uncommon cluster of findings: florid bilateral parotid disease, posterior uveitis, antecedent bilateral simultaneous peripheral facial palsy, calcitriol-driven hypercalcemia with bilateral obstructive nephrolithiasis, and a conspicuously silent chest. Taken together, they illustrate the diagnostic difficulty that extrapulmonary sarcoidosis can pose when the familiar radiographic clues are absent, and they frame the questions this report sets out to address.

### 3.1. Diagnostic Criteria and Phenotypic Variants of Heerfordt Syndrome

Heerfordt’s original description comprised a low-grade fever, parotid enlargement, anterior uveitis, and cranial neuropathy, most often peripheral facial-nerve palsy [3,4]. Subsequent reviews have made clear that complete forms are the exception rather than the rule, and most authors now accept an “incomplete” diagnosis when two of the four cardinal features coexist with compatible histological evidence of non-caseating granulomas [10]. Our patient fulfilled three of the four classical features (parotid enlargement, uveitis, and facial palsy) without overt fever. The posterior (rather than anterior) pattern of uveitis is somewhat atypical: the pooled case series report anterior involvement in roughly 60–70% of Heerfordt patients [10,11]. The cardinal features and accepted variants are summarized in Table 3. Importantly, IgG4-related disease is a key mimic of this overall presentation, since it can also cause bilateral salivary-gland enlargement, sicca symptoms, and tubulointerstitial renal disease; the finding of well-formed non-caseating granulomas on parotid biopsy—rather than the storiform fibrosis and IgG4-rich plasma-cell infiltrate that characterize IgG4-related disease—together with a markedly elevated serum ACE and PTH-independent, calcitriol-mediated hypercalcemia, favored sarcoidosis in our patient. Dedicated serum IgG4 measurement and IgG4 immunostaining of the biopsy were not performed during the admission; however, total serum IgG was within the reference range (12.20 g/L), and the biopsy showed well-formed non-caseating granulomas without the storiform fibrosis or obliterative phlebitis that typify IgG4-related disease, arguing against that diagnosis.

### 3.2. Bilateral Simultaneous Facial Palsy: A Clinical Red Flag

Bilateral simultaneous peripheral facial weakness (diplegia facialis) is uncommon, accounting for fewer than 2% of all lower-motor-neuron facial palsies [12,13]. Unlike unilateral Bell palsy, in which an idiopathic or post-viral etiology can be accepted with reasonable confidence after a brief workup, bilateral involvement carries a narrow but important differential. Leading causes include Lyme borreliosis, Guillain–Barré syndrome (especially the Miller Fisher and pharyngo-cervico-brachial variants), human immunodeficiency virus infection, leukemic or lymphomatous meningeal infiltration, basal meningitis (tuberculous, fungal, or carcinomatous), Möbius syndrome (in younger patients), and, relevant here, neurosarcoidosis [11,13]. Spontaneous recovery, as occurred in our patient, does not absolve the clinician of the obligation to search for an underlying systemic cause. The differential is summarized in Table 4.

### 3.3. Pathophysiology of Hypercalcemia in Sarcoidosis

Hypercalcemia complicates 5–10% of sarcoidosis cases, and hypercalciuria a considerably higher proportion, up to 40–60% in older biochemical surveys [14,15]. The mechanism is autonomous extrarenal synthesis of 1,25-dihydroxyvitamin D by 1α-hydroxylase (CYP27B1) expressed in granuloma-resident macrophages. The renal isoform of this enzyme is tightly regulated by PTH and FGF-23 and is suppressed by its own product; the granuloma-derived isoform behaves quite differently, remaining active even in the face of hypercalcemia and elevated calcitriol and escaping FGF-23 restraint. The result is unrestrained intestinal calcium absorption, mobilization of skeletal calcium, hypercalciuria, and, once the urinary calcium load exceeds tubular capacity, calcium-based nephrolithiasis [8,9]. Plasma PTH is appropriately suppressed, as it was in our patient (3.4 pg/mL). Consistent with substrate consumption by unregulated extrarenal 1α-hydroxylase rather than vitamin D excess, the 25-hydroxyvitamin D level was low-normal (22 ng/mL) despite frank hypercalcemia, and serum phosphate was normal to high-normal (4.40 mg/dL) rather than reduced, further distinguishing this PTH-independent, calcitriol-mediated pattern from PTH-driven hypercalcemia. The pathway is illustrated schematically in Figure 4.

### 3.4. The Renal Phenotype of Sarcoidosis

Renal involvement in sarcoidosis is heterogeneous. The prototypical histological lesion is granulomatous interstitial nephritis (GIN), characterized by non-caseating epithelioid granulomas with surrounding lymphoplasmacytic infiltration and variable interstitial fibrosis. Other recognized patterns include nephrocalcinosis, nephrolithiasis, distal renal tubular acidosis, nephrogenic diabetes insipidus and, much less often, membranous nephropathy, focal segmental glomerulosclerosis, and IgA nephropathy [6,7,16,17]. Acute kidney injury can arise from any of these mechanisms or their combination [18]. Obstructive uropathy from calcium-based stone disease, as in our patient, is mechanistically distinct from granulomatous inflammation of the interstitium, but the two coexist in a subset of patients [19]. Renal biopsy was deferred here because the metabolic and obstructive substrate adequately accounted for the AKI and the parotid biopsy had already established the underlying disease; subclinical granulomatous nephritis cannot be excluded with certainty, and longer-term renal surveillance is appropriate.

### 3.5. Comparison with Published Cases

A focused literature search of PubMed and Scopus (terms: “Heerfordt syndrome,” “uveoparotid fever,” “sarcoidosis hypercalcemia acute kidney injury,” “sarcoidosis nephrolithiasis”) returned 14 directly relevant English-language reports between 2010 and 2025. We compared our patient with eight of these in which sufficient clinical, biochemical, and outcome detail was available for structured comparison; the remaining six lacked adequate detail (Table 5). Several patterns emerge. First, bilateral simultaneous facial palsy is rare even within the Heerfordt subset; most published cases describe unilateral or sequential (“alternating”) involvement [10,11,20]. Second, hypercalcemia of the severity seen here is more often reported in isolation than as a component of the Heerfordt phenotype [16,17,19]. Third, the complete absence of any pulmonary radiographic finding is unusual: most published cases retain at least mediastinal adenopathy, apical change, or hilar prominence [21]. Fourth, where outcome data are available, the response to corticosteroid monotherapy is uniformly favorable, with renal recovery generally evident within two to four weeks, a pattern mirrored in our patient.

### 3.6. Therapeutic Considerations

Treatment of extrapulmonary sarcoidosis is largely empirical, derived from observational cohorts and expert consensus rather than randomized trials [22,23], and structured guidance specific to renal sarcoidosis remains limited [24]. For neurosarcoidosis or clinically significant renal involvement, oral prednisolone at 0.5–1 mg/kg/day for four to eight weeks, followed by a slow taper over six to twelve months, is widely used; pulse intravenous methylprednisolone is reserved for severe or rapidly progressive disease. Steroid-sparing agents (methotrexate, azathioprine, mycophenolate mofetil, or hydroxychloroquine) are introduced when relapse occurs on taper, when corticosteroid intolerance limits the dose, or when maintenance beyond 12 months is anticipated. Tumor necrosis factor inhibitors (infliximab, adalimumab) are reserved for refractory or organ-threatening disease [22].

In hypercalcemic patients, ultraviolet exposure and dietary calcium and vitamin D supplementation should be limited; bisphosphonates may be considered for severe acute hypercalcemia, but calcitriol-mediated hypercalcemia generally responds rapidly enough to corticosteroids that they are seldom necessary. Mechanical obstruction must be relieved promptly with ureteric stenting or percutaneous nephrostomy. Our patient illustrates this two-pronged approach: stenting addressed the mechanical problem while corticosteroids targeted the granulomatous substrate, and both interventions were needed for the rapid recovery observed.

### 3.7. Diagnostic Pitfalls and Learning Points

Several practical lessons deserve emphasis. Sarcoidosis should be considered in any patient with parathyroid-hormone-independent hypercalcemia regardless of the chest film, since exclusively extrathoracic disease accounts for missed or delayed diagnoses in an estimated 10–15% of cases [15]. Parotid biopsy is a high-yield, low-morbidity route to histological confirmation when superficial salivary disease is present, and obviates bronchoscopic sampling when the thorax is radiographically silent. Bilateral simultaneous facial palsy should never be ascribed by default to bilateral Bell palsy; the differential is narrow and warrants a structured workup. Normal serum ACE does not exclude sarcoidosis, but markedly elevated levels in the appropriate clinical setting support the diagnosis and can serve as a coarse marker of disease activity [25]. Finally, the speed of biochemical and clinical response to corticosteroids in sarcoidosis-related AKI argues for early empirical treatment once competing diagnoses, notably tuberculosis and malignancy, have been reasonably excluded.

**Key Teaching Points**

- PTH-independent hypercalcemia with elevated ACE in a young adult should prompt active investigation for sarcoidosis, even when the chest film is normal.
- Bilateral simultaneous peripheral facial palsy is a clinical red flag with a narrow differential; neurosarcoidosis should always be on the list.
- Posterior uveitis can occur in Heerfordt syndrome and does not exclude the diagnosis; anterior involvement is more common but not obligatory.
- Parotid biopsy is high-yield when accessible disease is present and avoids more invasive bronchoscopic sampling in radiographically silent chests.
- Sarcoidosis-related AKI is largely reversible when both the mechanical (stenting) and granulomatous (corticosteroid) substrates are addressed in parallel.
- Calcitriol-mediated hypercalcemia responds to glucocorticoids, which inhibit macrophage 1α-hydroxylase activity; bisphosphonates are seldom required.

### 3.8. Limitations

Several limitations should be acknowledged. Active vitamin D (1,25-dihydroxyvitamin D) could not be measured during admission because the assay was unavailable at our institution, which would have provided direct biochemical confirmation of the proposed mechanism of hypercalcemia. Renal biopsy was not performed, since the obstructive and metabolic substrate accounted for the acute kidney injury and a histological diagnosis had already been secured at the parotid; subclinical granulomatous interstitial nephritis therefore cannot be formally excluded. A 24 h urinary calcium measurement and a spot urine albumin-to-creatinine ratio were not obtained (24 h urinary protein was quantified instead, at 595.9 mg), so the magnitude of hypercalciuria could not be documented directly; serum calcium was used to monitor the metabolic response and normalized within a week of starting corticosteroids, remaining normal at the three-month review. Long-term follow-up beyond three months is ongoing and will be reported separately if clinically meaningful changes arise.

## 4. Conclusions

We have described a young man with Heerfordt syndrome whose clinical picture was dominated by extrathoracic complications: severe parathyroid-hormone-independent hypercalcemia, bilateral upper-ureteric calculi, and obstructive acute kidney injury, all on a background of antecedent bilateral simultaneous peripheral facial palsy and posterior uveitis, all in the absence of radiographic thoracic disease. To our knowledge this particular constellation has not previously been reported. The patient recovered fully after ureteric decompression and oral corticosteroid therapy. The case argues for an active search for sarcoidosis in patients with otherwise unexplained calcium dysregulation, parotid swelling, or bilateral facial weakness regardless of the chest film, and reinforces that sarcoidosis-related renal injury is largely reversible when treated promptly. Wider clinician familiarity with these less common phenotypes will reduce diagnostic delay and the attendant risk of irreversible end-organ damage.

## Figures and Tables

**Figure 1:**
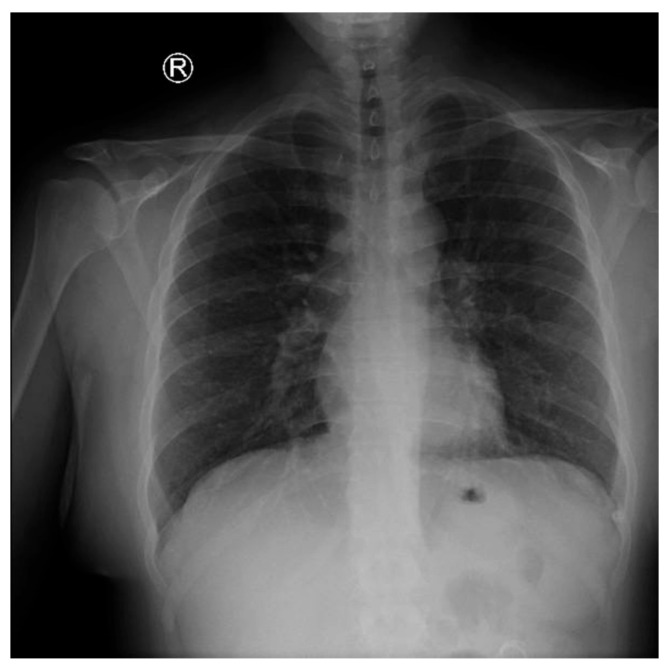
Posteroanterior chest radiograph at presentation. The lung fields are clear bilaterally with no infiltrate, the cardiomediastinal silhouette is normal, and there is no bilateral hilar lymphadenopathy or parenchymal disease. The absence of any thoracic abnormality, which is uncommon in sarcoidosis, delayed early consideration of the diagnosis and underscores the importance of pursuing extrapulmonary manifestations on their own clinical merits. The circled letter “R” is a radiographic laterality marker denoting the patient’s right side.

**Figure 2:**
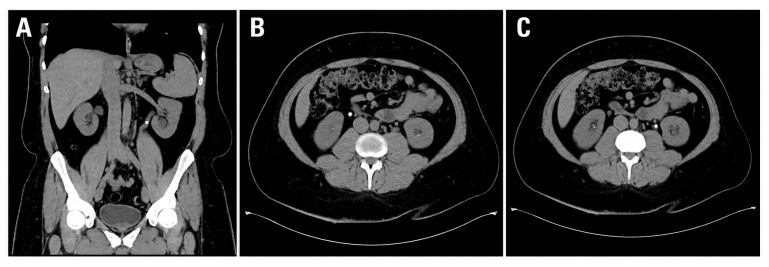
Non-contrast computed tomography of the abdomen and pelvis. (**A**) Coronal reconstruction showing both kidneys with bilateral intrarenal calcifications (hyperdense foci within the renal collecting systems) and mild pelvicalyceal fullness. (**B**,**C**) Representative axial images demonstrating the bilateral upper-ureteric calculi (8 mm on the right and 6 mm on the left) together with small non-obstructing renal calculi. The radiographic pattern reflects sustained calcitriol-driven hypercalciuria.

**Figure 3:**
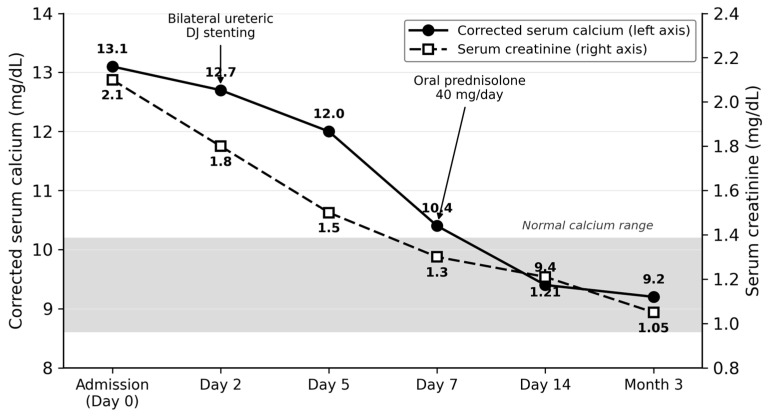
Clinical course. Trends in corrected serum calcium (left axis) and serum creatinine (right axis) from admission to the three-month review, with timing of the principal interventions indicated. The shaded band marks the normal calcium reference range (8.6–10.2 mg/dL). Note the rapid biochemical improvement after introduction of oral prednisolone. DJ, double-J ureteric stent.

**Figure 4:**
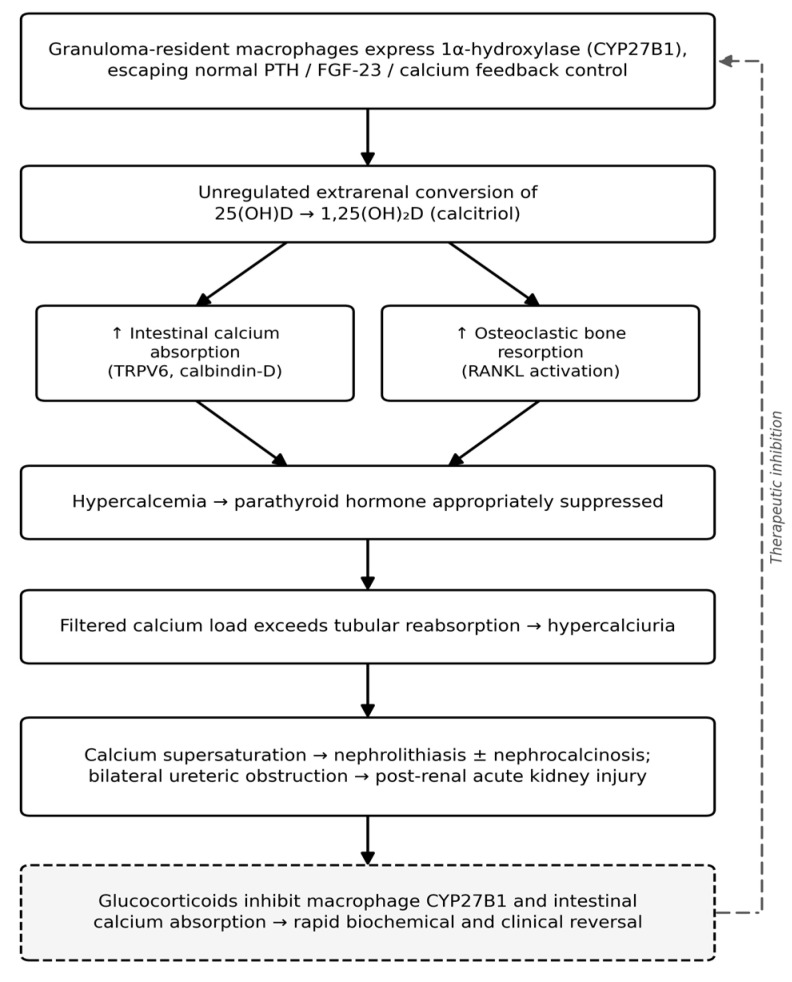
Pathophysiology of calcitriol-mediated hypercalcemia in sarcoidosis (schematic). Granuloma-resident macrophages generate 1,25-dihydroxyvitamin D through feedback-independent 1α-hydroxylase activity, driving intestinal calcium absorption and bone resorption; the consequent hypercalcemia and hypercalciuria promote nephrolithiasis, obstruction, and post-renal acute kidney injury. Glucocorticoids inhibit macrophage CYP27B1, accounting for the rapid reversal observed with treatment. Solid arrows indicate the direction of the pathophysiological sequence, and the dashed arrow denotes the site of therapeutic (glucocorticoid) inhibition. 25(OH)D, 25-hydroxyvitamin D; 1,25(OH)_2_D, 1,25-dihydroxyvitamin D (calcitriol); RANKL, receptor activator of nuclear factor-κB ligand; TRPV6, transient receptor potential vanilloid 6.

**Table 1:** Biochemistry, renal function, and metabolic panel at admission.

Parameter	Result	Reference Range	Interpretation
Creatinine (admission)	2.1 mg/dL	0.67–1.17 mg/dL	Elevated (AKI)
Creatinine (post-treatment)	1.21 mg/dL	0.67–1.17 mg/dL	Improving
Urea	64 mg/dL	19–44 mg/dL	Elevated
Uric acid	9.9 mg/dL	3.4–7.0 mg/dL	Elevated
Total calcium (admission)	13.1 mg/dL	8.6–10.2 mg/dL	Severely elevated
Total calcium (post-treatment)	9.4 mg/dL	8.6–10.2 mg/dL	Normal
Phosphate	4.40 mg/dL	2.5–4.5 mg/dL	Normal
Magnesium	1.90 mg/dL	1.7–2.2 mg/dL	Normal
Intact PTH	3.4 pg/mL	15–65 pg/mL	Suppressed
25-hydroxyvitamin D	22 ng/mL	30–100 ng/mL	Insufficient
1,25-dihydroxyvitamin D	Not assayed	—	Unavailable at site
Angiotensin-converting enzyme	>100 U/L	8–52 U/L	Elevated
TSH/free T4	Within range	—	Euthyroid
Albumin	4.7 g/dL	3.5–5.2 g/dL	Normal
Total protein	7.4 g/dL	6.0–8.0 g/dL	Normal
AST/ALT/ALP/GGT	Within range	—	Normal liver panel
Sodium	138 mmol/L	136–145 mmol/L	Normal
Potassium	4.63 mmol/L	3.5–5.0 mmol/L	Normal
Chloride	108 mmol/L	98–106 mmol/L	Mildly elevated
Fasting glucose/HbA1c	76 mg/dL/4.97%	—	Normal
Lipid panel	LDL 112, HDL 29	—	Low HDL
LDH	172 U/L	140–280 U/L	Normal

Abbreviations: AKI, acute kidney injury; PTH, parathyroid hormone; TSH, thyroid-stimulating hormone.

**Table 2:** Hematology, immunology, infectious workup, and urinalysis.

Parameter	Result	Interpretation
Hemoglobin/Hematocrit	14.2 g/dL/43.6%	Normal
WBC count (differential)	8.7 × 10^9^/L; lymphocytes 12.8%	Lymphopenia
Platelets	418 × 10^9^/L	Mild thrombocytosis
PT/INR/APTT	Within range	Normal
ESR/CRP	19 mm/h/10 mg/L	Mildly elevated
C3/C4	1.61 g/L/0.332 g/L	Normal
IgG	12.20 g/L	Normal
ANA/c-ANCA/p-ANCA	Negative	Negative
Anti-Ro (SSA)/Anti-La (SSB)	Negative	Negative
HIV/HBsAg/HCV antibody	Non-reactive	Negative
IGRA (M. tuberculosis)	Non-reactive	Negative
24 h urinary protein	595.9 mg/24 h	Sub-nephrotic proteinuria
Urine leukocytes	>100/HPF	Sterile pyuria
Urine red blood cells	3+	Microscopic hematuria
Urine protein (dipstick)	2+	Proteinuria
Hyaline casts	3+	Present
Urine culture (×4)	No growth	Sterile

Abbreviations: ANA, antinuclear antibody; ANCA, antineutrophil cytoplasmic antibody; HPF, high-power field; IGRA, interferon-gamma release assay. Urine findings are reported semi-quantitatively; the plus sign (e.g., 2+, 3+) denotes the dipstick/microscopy grade of positivity.

**Table 3:** Cardinal features and accepted variants of Heerfordt syndrome.

Feature	Classical Description	Reported Variants	Frequency in Published Series
Parotid enlargement	Bilateral, painful, persistent	Unilateral; submandibular involvement	~80–95%
Uveitis	Anterior, granulomatous	Posterior uveitis; panuveitis; vitritis	~70–80%
Facial nerve palsy	Unilateral, peripheral	Bilateral simultaneous; alternating; recurrent	~50–60%
Fever	Low-grade, intermittent	Absent in up to 40% of contemporary reports	~50–60%
Complete tetrad	All four features	Two of four with biopsy support is accepted	~10–15%

Reported frequencies are pooled estimates from major case series and reviews [4,10,11]; precise figures vary because of small numbers and selection bias.

**Table 4:** Differential diagnosis of bilateral simultaneous peripheral facial palsy.

Category	Specific Entity	Key Clues/Investigations
Infectious	Lyme borreliosis	Erythema migrans; tick exposure; serology
Infectious	HIV (acute or advanced)	Risk factors; ELISA/Western blot
Infectious	Tuberculous or fungal meningitis	Imaging; CSF analysis; cultures
Infectious	Otitis/parotitis/Ramsay Hunt	Local examination; vesicular rash
Inflammatory	Sarcoidosis (Heerfordt or pure neurosarcoidosis)	ACE; calcium; chest imaging; biopsy
Inflammatory	Sjögren syndrome	Sicca symptoms; anti-Ro/anti-La
Neurological	Guillain–Barré (incl. Miller Fisher)	Areflexia; albumin–cytological dissociation
Neoplastic	Leukemic/lymphomatous infiltration	Smear; LDH; bone marrow; CSF cytology
Neoplastic	Meningeal carcinomatosis	MRI with contrast; CSF cytology
Vascular/structural	Brainstem ischemia/pontine lesion	MRI brain
Congenital	Möbius syndrome	Onset in infancy; abducens involvement
Idiopathic	Bilateral Bell palsy (diagnosis of exclusion)	<25% of bilateral cases; exhaustive exclusion

Adapted from Keane [12] and Jha et al. [13]. ACE, angiotensin-converting enzyme; CSF, cerebrospinal fluid; LDH, lactate dehydrogenase; MRI, magnetic resonance imaging.

**Table 5:** Comparison of the present case with published reports of Heerfordt syndrome and sarcoidosis-related hypercalcemia/acute kidney injury.

Feature	Present Case	Chappity et al. [10]	Sharma et al. [16]	Sharmeen et al. [17]	Fujiwara et al. [21]	Denny et al. [20]	Zakharova et al. [19]	Gallo et al. [11]
Age/sex	30/M	52/F	61/F	55/M	53/M + 55/F	59/F	Not specified	47/F
Heerfordt syndrome	Complete (3/4)	Complete	Absent	Absent	Incomplete + complete	Complete	Absent	Partial
Parotid involvement	Bilateral	Bilateral	No	No	Unilateral/bilateral	Bilateral	No	No
Facial palsy	Bilateral simultaneous	Bilateral alternating	No	No	Unilateral	Unilateral	No	Bilateral simultaneous
Uveitis	Posterior, bilateral	Anterior	No	No	Absent/present	Anterior	No	Absent
Hypercalcemia	Severe (13.1)	No	Severe	Severe	No	No	Severe	No
PTH	Suppressed (3.4)	Normal	Suppressed	Suppressed	—	—	Suppressed	—
ACE	Elevated (>100)	Elevated (129)	Elevated	Elevated	Elevated	Elevated	Elevated	Elevated
Renal involvement	Obstructive AKI	None	AKI	GIN + AKI	None	None	GIN + stones + AKI	None
Nephrolithiasis	Bilateral ureteric	No	No	Yes (history)	No	No	Yes	No
Thoracic imaging	Normal	Mediastinal LAD	Apical bronchiectasis	Mediastinal LAD	Mediastinal LAD	Hilar markings	Not detailed	BHL
Biopsy site	Parotid	Parotid	Marrow + lung	Kidney (GIN)	Eyelid + node	Lymph node	Kidney	Lymph node
Dialysis required	No	No	No	No	No	No	Yes (transient)	No
Steroid response	Excellent	Excellent	Good	Good	Excellent	Excellent	Good	Good

ACE, angiotensin-converting enzyme; AKI, acute kidney injury; BHL, bilateral hilar lymphadenopathy; F, female; GIN, granulomatous interstitial nephritis; LAD, lymphadenopathy; M, male; PTH, parathyroid hormone.

## Data Availability

The data supporting the findings of this report are contained within the article. Additional anonymized data are available from the corresponding author on reasonable request, subject to institutional confidentiality requirements.
